# Rock Particle Motion Information Detection Based on Video Instance Segmentation

**DOI:** 10.3390/s21124108

**Published:** 2021-06-15

**Authors:** Man Chen, Maojun Li, Yiwei Li, Wukun Yi

**Affiliations:** 1 School of Electrical and Information Engineering, Changsha University of Science and Technology, Changsha 410114, China; 19205060770@stu.csust.edu.cn (M.C.); lywlyw@stu.csust.edu.cn (Y.L.); 2 Xiaoxiang Research Institute of Big Data, Changsha 410199, China; ywk@xxribd.org.cn

**Keywords:** rock particles, machine vision, video instance segmentation, motion information detection

## Abstract

The detection of rock particle motion information is the basis for revealing particle motion laws and quantitative analysis. Such a task is crucial in guiding engineering construction, preventing geological disasters, and verifying numerical models of particles. We propose a machine vision method based on video instance segmentation (VIS) to address the motion information detection problem in rock particles under a vibration load. First, we designed a classification loss function based on Arcface loss to improve the Mask R-CNN. This loss function introduces an angular distance based on SoftMax loss that distinguishes the objects and backgrounds with higher similarity. Second, this method combines the abovementioned Mask R-CNN and Deep Simple Online and Real-time Tracking (Deep SORT) to perform rock particle detection, segmentation, and tracking. Third, we utilized the equivalent ellipse characterization method for segmented particles, integrating with the proportional calibration algorithm to test the translation and detecting the rotation by calculating the change in the angle of the ellipse’s major axis. The experimental results show that the improved Mask R-CNN obtains an accuracy of 93.36% on a self-created dataset and also has some advantages on public datasets. Combining the improved Mask R-CNN and Deep SORT could fulfill the VIS with a low ID switching rate while successfully detecting movement information. The average detection errors of translation and rotation are 5.10% and 14.49%, respectively. This study provides an intelligent scheme for detecting movement information of rock particles.

## 1. Introduction

Rock particles are common in geological disasters and are a type of material that is widely adopted in construction and transportation. The development of a general theory of the motion of granular materials is one of the 125 scientific frontier issues of *Science*. Revealing the movement laws and quantitatively analyzing rock particles in a moving state can provide reasonable guidance for the construction process and to improve construction efficiency [1]. It can also offer meso-analysis related to the occurrence of geological disasters, which can assist in their prevention [2,3]. In addition, with the research and application of numerical simulation methods (e.g., finite element method and discrete element method) in geotechnical engineering, the lack of effective verification data is a principal reason for restricting their development [4,5,6]. The motion information detection of rock particles not only is the basis of revealing their motion laws and quantitative analysis, but also can provide reliable data for particle numerical models, which is of great value in engineering and scientific research [7].

The detection of particle motion information is done using video instance segmentation (VIS). This means detecting, segmenting, and tracking instances simultaneously [8]. It is more challenging than image instance segmentation in that it not only requires instance segmentation on individual frames, but also the tracking of instances across frames.

In this study, we focused on detecting the motion information of rock particles under a vibration load and proposed a VIS-based method. This method combines the current mainstream instance segmentation and object tracking algorithms. Experimental equipment was established to verify the proposed method, and the effectiveness of our technique was demonstrated through experiments. We summarize them as follows:

1. Because the objects and backgrounds have a high degree of similarity, we improved the Mask R-CNN to develop the feature recognition ability and the instance segmentation effect of particles by designing a classification loss function based on Arcface loss.
2. The multiparticle VIS with a low ID switching rate was achieved by combining the improved Mask R-CNN and SORT.
3. We utilized the equivalent ellipse characterization method to process the segmented particles, combining characterization results with the proportional calibration algorithm to detect its translation and detecting the rotation by calculating the change in the angle of the major axis of the ellipse.
4. We verified the effectiveness of the method through segmentation experiments, VIS, and motion information detection. The experimental results showed that the improved Mask R-CNN had a better detection and segmentation effect. The ID switching rate of the VIS was low. This method can successfully detect the movement information of rock particles. The average detection errors of translation and rotation are 5.10% and 14.49%, respectively.

## 2. Related Work

Few studies on particle VIS have been conducted. However, particle segmentation as the basis of VIS has been extensively researched with the development of machine vision. In addition, there is substantial work on image-based deformation and stress measurement.

### 2.1. Traditional Particle Segmentation Method

The main traditional methods have relied on regional information and then combined with related traditional algorithms to accomplish particle segmentation. With the operation of edge connection, Yen et al. [9] used the Canny edge detection algorithm to detect particle images so that the edge gaps were changed to multiple closed regions. Zhang et al. [10] combined the bi-windows and maximum between-class variance (OSTU) to obtain binary images. Then, the distance transformation principle was employed to obtain the best seed area with a higher gray value, and a Watershed algorithm based on markers was applied to segment the particles. Amankwah et al. [11] proposed a method for segmenting particles, which utilized the mean shift algorithm to identify pixel clusters of particular modes from the probability density function of the image data. The pixel clusters were then used to generate markers for the watershed transform and shadow areas in images. However, the particle images often have characteristics of density. The abovementioned methods cannot accurately extract particle features, and the segmentation over-relies on manual selection of features.

### 2.2. Particle Segmentation Method Based on Deep Learning

Deep learning [12] has several advantages in image processing and, therefore, has been used in particle segmentation tasks. Yuan et al. [13] focused on solving the inaccuracy problem caused by mutual adhesion and shadows in the ore images using advanced ore image segmentation method based on the holistically-nested edge detection method. Compared with the traditional algorithm, their proposed method was more robust. Duan et al. [14] designed a lightweight U-net deep learning network to automatically detect particles from images and to obtain the probability maps of particle contours. This method could be applied to particle product quality monitoring. Liu et al. [15] proposed an image segmentation method based on the U-net and Res-Unet networks. The proposed method preprocessed the original images captured from an open-pit mine to reduce noise and extract the object region with grayscale, median filter, and adaptive histogram using equalization techniques. The abovementioned deep learning methods are mainly used for the semantic segmentation of particles that can automatically realize particle feature extraction with higher accuracy. These methods have become the main research direction for particle segmentation. However, because semantic segmentation can only acquire the complete information of all particles, it is difficult to analyze the meso-mechanism on a single-particle scale.

### 2.3. The Image-Based Measurement of Deformation and Stresses

Over the years, many visual sensors have been used in the monitoring and prediction of natural disasters (e.g., landslides, slope failure, and rockfalls) because of their low cost compared to expensive terrestrial laser scanner (TLS) or Interferometric Synthetic Aperture Radar (InSAR) [16,17]. Concurrently, image-based measurement of deformation and stresses were also being studied extensively. The most widely known method is particle image velocimetry (PIV) [18]. The PIV can combine a range of advances in image analysis algorithms (e.g., image intensity interpolation [19] and deformation parameter optimization [20]) that are best suited to geotechnical applications, which would be faster and more precise than the standard PIV approach [21]. However, these image-based methods could not analyze challenging scenes, such as images with insufficient lighting and fast-moving objects [22]. However, many related complementary methods have been studied and applied. Gance et al. [23] proposed a target detection and tracking (TDT) method that was based on simple binary image processing for the analyses of long time series. This method can be used as a complement to image correlation and other displacement observation methods for rapid assessments. In terms of moving-object tracking methods, feature tracking, color feature tracking, and outline tracking have proven to be effective tracking methods [24]. However, 3D reconstruction technology has some advantages compared to feature tracking because it also approximates the 3D shape of an object using a 3D mesh. Guccione et al. [25] used four high-speed cameras and two tilted mirrors for 3D reconstruction to track the objects. Then, the 3D rotational velocities were estimated accurately using a new postprocessing algorithm. The abovementioned devices and methods have made important contributions to meso-analysis and have helped in preventing geological disasters.

## 3. Motion Information Detection of Rock Particles Based on VIS

### 3.1. Method Framework

**Figure 1:**
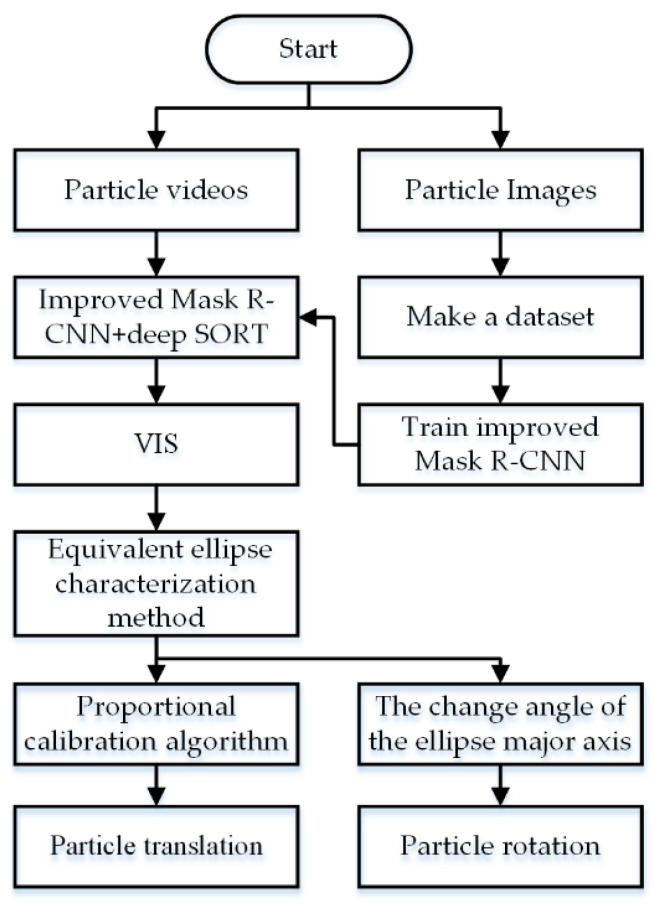
Method framework.

Because of the complexity of detecting the motion information of rock particles, it is necessary to segment the video instance first. For resiliency in environments in which the objects and backgrounds are highly similar, we designed a new classification loss function for Mask R-CNN based on Arcface loss to improve the feature recognition ability and ameliorate the instance segmentation effect of particles. The combination of the improved Mask R-CNN and Deep SORT can further accomplish the VIS of rock particles. The use of the equivalent ellipse characterization method can extract the center position of each particle frame by frame, and the pixel translation can be obtained by subtracting the center position of the previous frame. Then, the proportional calibration algorithm can calibrate the pixel translation and actual translation. On the other hand, the rotation can be detected by calculating the change in the angle of the ellipse’s major axis. The overall algorithm framework is illustrated in Figure 1.

### 3.2. Particle Segmentation

#### 3.2.1. Mask R-CNN

**Figure 2:**
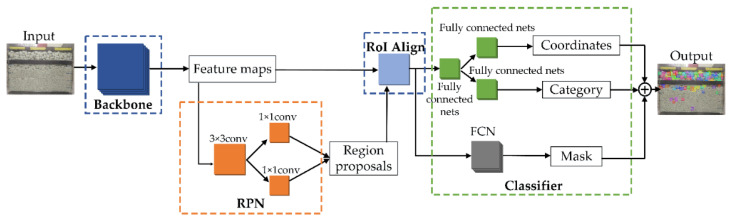
Mask R-CNN network structure.

Mask R-CNN [26] adds a mask prediction branch based on Faster R-CNN [27] and achieves pixel-level segmentation of images by combining the advantages of object detection and semantic segmentation networks. It has been used in fields such as agriculture, engineering, and daily life [28,29,30]. The region of interest alignment (RoI Align) is exploited to replace the region of interest pooling (RoI pooling) in Mask R-CNN, which solves the region mismatch problem. The overall network structure of Mask R-CNN is shown in Figure 2. It is composed mainly of four parts: backbone, region proposal network (RPN), RoI Align, and classifier.

The backbone is a series of convolutional layers that can extract the feature maps. In this study, we chose ResNet-101 as the backbone. ResNet-101 had five convolutional layers. The samples were convolved, regularized, and activated in each layer to obtain feature maps of different sizes. Then, the use of feature pyramid networks (FPNs) [31] could fuse multilayered semantic features and acquire feature maps after fusion of various sizes.

The RPN is a network that can extract region proposals through classification and regression. The inputs of the RPN are the outputs of the last layer of the FPN. First, a certain number of anchors are generated for each pixel of the feature maps. The likelihood that these anchors are the foreground or background and the offset between these anchors and their corresponding ground truth is calculated. Then, the loss of classification and regression could be assessed according to the loss function of the RPN.

RoI Align utilizes bilinear interpolation to replace the quantization rounding operation of RoI pooling. It can convert the region proposals into fixed-size feature maps without losing spatial information so that each pixel can maintain accurate coordinates.

The classifier had three parallel branches. Two of these are categories and coordinates that can obtain a precisely positioned bounding box. The other branch utilizes a fully convolutional network (FCN) [32] to predict the mask and map the feature size to the image size.

#### 3.2.2. Classification Loss Function Based on Arcface Loss

The rock particles were densely distributed and are in close contact with each other in the experimental environment. Considering the task of extracting the translation and rotation of particles in this study, we attributed the coarse particles that were heavily obscured as the background for reducing the interference of such coarse particles on the accuracy of motion information. The characteristics of such particles in the background were similar to those of the objects. In addition, many small particles in the background were similar to the objects. The diameters of the coarse and small particles were 20–30 mm and 2.5–7.5 mm, respectively, and the diameter ratio of coarse particles to small particles was about 2.7–12. These two types of particles (coarse particles that are heavily occluded and small particles) in the background were highly similar to the object particles.

SoftMax [33] loss is usually used to calculate the possibility and ensure the separability of objects as a common classification loss function in an object detection model. As many particles are heavily obscured and small in the experimental environment, it is difficult to distinguish features when using the traditional SoftMax loss. The A-SoftMax loss [34], which is based on the traditional SoftMax loss, was used to improve the feature discrimination ability of the model. It adds the angular distance based on SoftMax loss, which can distinguish the objects and backgrounds with higher similarity. The A-SoftMax loss can be expressed as follows:

(1) $$L_{cls}=\frac{1}{N}\sum_{i=1}^{N}\log(\frac{e^{\left\|x_{i}\right\|\varphi (\theta_{y_{i}},i)}}{e^{\left\|x_{i}\right\|\varphi (\theta_{y_{i}},i)}+\sum_{j\neq y_{i}}e^{\left\|x_{i}\right\|\varphi (\theta_{j},i)}})$$

(2) $$\varphi (\theta_{y_{i}},i)={\left(-1\right)}^{k}\cos(m\theta_{y_{i}},i)-2k$$

where *N* is the number of training samples, *x_i_* is the quantitative data of object features, $\theta_{y_{i}}$ is the angle between the object feature vector and *X_i_* weight $W_{y_{i}}$, $\theta_{y_{i}}\in [\frac{k\pi }{m},\frac{(k+1)\pi }{m}]$, k∈[0,m−1], and *m* is the angular magnification.

The above loss function can narrow the within-class distance of the network model and enlarge the between-class distance, which can improve the feature classification ability of the model. However, the angle amplification mechanism also makes it difficult for the model to converge. Therefore, we used a classification loss function based on Arcface loss [35] by integrating the features of SoftMax loss, which can be expressed as follows:

(3) $$L_{cls}=-\frac{1}{N}\sum_{i=1}^{N}\log(\frac{e^{s(\cos(\theta_{y_{i}}+n))}}{e^{s(\cos(\theta_{y_{i}}+n))}+\sum_{j\neq y_{i}}e^{s\cos(\theta_{j})}}),$$

where *s* is the fixed scale factor and *n* is the angle margin.

**Figure 3:**
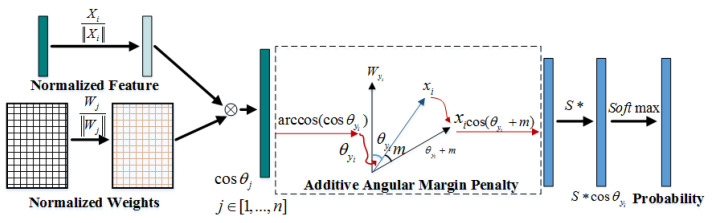
Arcface loss theory. * indicates multiplication.

The theory of Arcface loss is shown in Figure 3. *X_i_* is feature and *W* is the ground truth weight. Based on *X_i_* and *W* normalization, we could get $\cos\theta_{j}$. The inverse cosine function can calculate the angle between *X_i_* and $W_{y_{i}}$. The cosine distance can be obtained by adding *n* the object angle. Then, it outputs to the SoftMax layer after multiplying with *s*. Compared with A-SoftMax loss, Arcface loss utilizes angular distance and abandons the angle magnification mechanism to make the network converge more easily.

The other two parts of loss function are the regression loss function $L_{reg}$ and mask loss function $L_{mask}$. $L_{reg}$ obtains the bounding-box regression loss by the smooth function and $L_{mask}$ is the binary cross entropy loss function. Therefore, the overall loss function can be expressed as follows:

(4) $$L=L_{cls}+L_{reg}+L_{mask}$$

where $L_{reg}$ and $L_{mask}$ are shown in the equations below:

(5) $$L_{reg}=smooth(t_{i}+t_{i}^{\prime })=\left\{ \begin{array}{l} 0.5x,if\left|t_{i}-t_{i}^{\prime }\right|<1\\ \left|t_{i}-t_{i}^{\prime }\right|-0.5,otherwise \end{array}\right.,$$

(6) $$L_{mask}=-\sum_{i}p_{i}\log(p_{i}^{\prime })+(1-p_{i})(1-p_{i}^{\prime }),$$

where $t_{i}$ is the vector with four-coordinate data of the bounding box and $t_{i}^{\prime }$ is the vector with the data of the ground truth. $p_{i}$ is the binary classification probability and $p_{i}^{\prime }$ is the *i-th* output.

### 3.3. Particle Tracking

**Figure 4:**
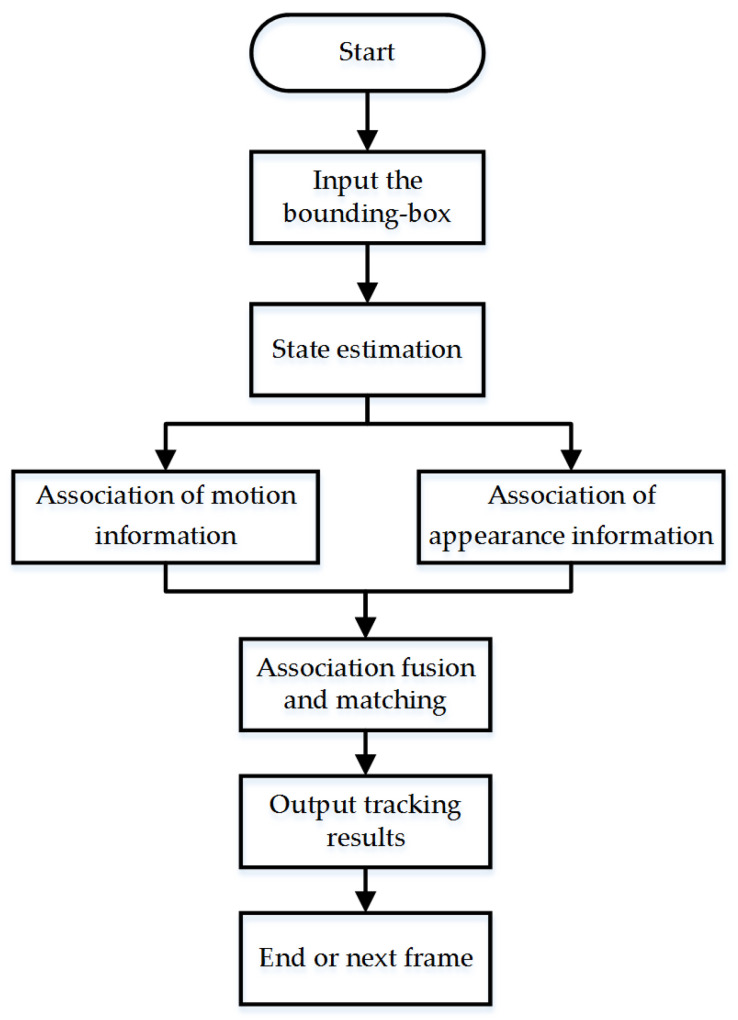
Deep SORT tracking framework.

As an improvement of SORT [36], Deep SORT [37] adds appearance information to matching problems, which can reduce the switching of object IDs. It is divided mainly into three aspects: state estimation, trajectory processing, and matching problems. The main tracking framework is shown in Figure 4.

The state of the trajectory at a certain moment is represented by an eight-dimensional space of $\left(u,v,r,h,\overset{\cdot }{u},\overset{\cdot }{v},\overset{\cdot }{r},\overset{\cdot }{h}\right)$, where (u,v) is the center position of the bounding box, *r* is the ratio of length to width, *h* is the height, and the other four variables represent the speed information in the image coordinates. A standard Kalman filter [38] based on a constant-velocity model and a linear observation model can predict the motion state. The predicted result was (u,v,r,h).

Trajectory processing consists of two parts: new detection result processing and tracker processing. If the detection result cannot be associated with the existing trajectory, it is regarded as a new detection result, and a new trajectory hypothesis is applied. If the new trajectory can be successfully associated in the first three frames, it is determined as a new object tracking. Otherwise, the trajectory was deleted. On the other hand, we set up a tracker that increases progressively during Kalman filter prediction and sets it to 0. If the tracker exceeds the predefined maximum threshold $A_{\max}$, we consider that object tracking is over.

The Deep SORT considers the association of motion information and appearance information simultaneously and combines them to calculate the degree of matching between the detection results and trajectories.

Deep SORT takes advantage of the Mahalanobis distance to describe the degree of motion information association. The Mahalanobis distance reflects the uncertainty of the state measurement by calculating the standard deviation between the detected position and the average tracking position. This can be expressed as follows:

(7) $$d^{\left(1\right)}(i,j)={\left(d_{j}-y_{i}\right)}^{T}S_{i}^{-1}(d_{j}-y_{i}),$$

where $d_{j}$ represents the position of the detected bounding box *j-th*, $y_{i}$ represents the predicted position of the object by the tracker, and $S_{i}$ is the covariance matrix between the detection position and average tracking position. In addition, Deep SORT excludes impossible associations by Mahalanobis distance thresholding within the 95% confidence interval calculated from the inverse distribution $\chi^{2}$. This can be expressed as follows:

(8) $$b_{i,j}^{\left(1\right)}=1[d^{\left(1\right)}(i,j)\leq t^{\left(1\right)}]$$

where $t^{\left(1\right)}=9.4877$. If the Mahalanobis distance is less than the specified threshold $t^{\left(1\right)}$, the motion state will be successfully associated.

In terms of the appearance information association of objects, Deep SORT obtains the 128-dimensional feature vector of $r_{j}$ the bounding box through $d_{j}$ the CNN network. The restriction condition was $\left\|r_{i}\right\|=1$. For each trajectory k, a library of $R_{k}$ appearance descriptors is applied to store the feature vector of the last 100 frames. Finally, the minimum cosine distance between the trajectory *i-th* and trajectory *j-th* can be calculated [39]. This process can be expressed as follows:

(9) $$d^{\left(2\right)}(i,j)=\min\left\{1-r_{j}^{T}r_{k}^{\left(i\right)}|r_{k}^{\left(i\right)}\in R\right\}$$

Similarly, Deep SORT sets a threshold $t^{\left(2\right)}$ for this information association, which can be expressed as follows:

(10) $$b_{i,j}^{\left(2\right)}=1[d^{\left(2\right)}(i,j)\leq t^{\left(2\right)}]$$

The linear weights of the two association methods are utilized to reflect the final degree of association, which can be expressed as follows:

(11) $$c_{i,j}=\lambda d^{\left(1\right)}(i,j)+(1-\lambda )d^{\left(2\right)}(i,j)$$

where λ is the associated weight of the motion information.

### 3.4. Motion Information Detection

Translation and rotation are the most representative motion information parameters in engineering and scientific research. It is necessary to characterize the particles to detect the translation and rotation of rock particles under a vibration load. Common characterization methods include the smallest circumscribed circle, the smallest circumscribed rectangle, and the equivalent ellipse [40]. We chose the equivalent ellipse as the characterization method for the detection of the translation and rotation of particles on account of the appearance characteristics of the rock particles.

**Figure 5:**
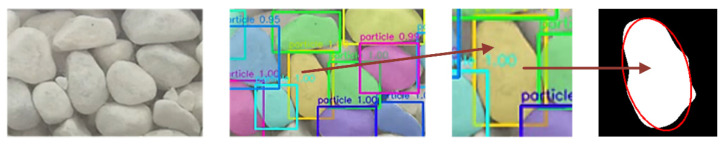
Equivalent ellipse characterization method.

We detected and segmented the masks of particles using the improved Mask R-CNN and tracked these masks with Deep SORT. Then, these masks were processed by the equivalent ellipse characterization method, which contributed to converting them into a numerical value with practical meaning, as shown in Figure 5.

The centers of the ellipses could replace the centers of mass of the particles. We extracted the center position of the ellipse frame by frame and obtained the translation by subtracting the center position of the previous frame. The units of the results measured by the above method are pixels. To acquire the actual translation of the rock particles, calculating the physical size of a single pixel is necessary, which is the calibration of image pixels [41]. We calibrated the images using a proportional calibration algorithm, which can be expressed as follows:

(12) $$k=\frac{L}{N}$$

where *k* is the actual size of pixels, *L* is the actual size, and *N* is the number of pixels. The rotation can be acquired by calculating the change in the major axis angles of the ellipses frame by frame.

## 4. Experiment and Analysis

### 4.1. Experimental Equipment and Parameter Settings

**Figure 6:**
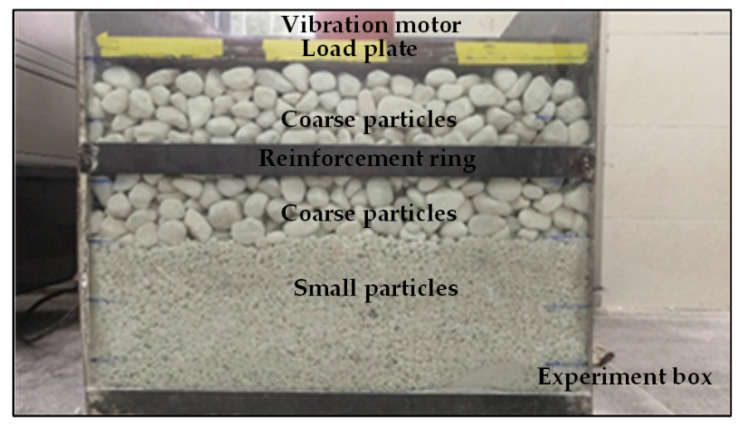
Experimental equipment.

We developed experimental equipment to collect videos, including the experiment box, vibration motor, load plate, reinforcement ring, CCD camera, transparent panel, coarse particles, and small particles. In this experiment, the vibration motor acted on the load plate and provided a vibration load for the particles; the reinforcement ring was used to fix the experiment box; the transparent panel could block particles; the CCD camera captured videos of the experimental environment. The SHL-500W CCD camera had a resolution of 1600 × 1200 pixels and a frame rate of 30 fps. The lens model used was LT-C0516-5MP with a focal length of 5 mm. The horizontal distance between the experiment box and the CCD camera was 143 cm, and the bottom width of the experiment box was 840 mm. The particles selected for this experiment were pebbles with irregular shapes and smooth surfaces. An image of the experimental environment captured by the visual sensor is shown in Figure 6.

The training and testing experiments of the model were conducted on Ubuntu 18.04. The processor was an Intel Core i7-8700K CPU @3.7 GHz (Intel, Mountain View, CA, USA), and the GPU was an NVIDIA GeForce RTX2080Ti (NVIDIA, Santa Clara, CA, USA). The framework of the deep learning framework was TensorFlow. The official weight was utilized as the pretraining weight, and the stochastic gradient descent was used during network training to accelerate the convergence speed of the Mask R-CNN. We set 80 epochs and performed 100 iterations per epoch; therefore, a total of 8 × 10^3^ iterations were performed. The activation function was ReLU, and the batch size was 2. The other key training parameters are listed in Table 1.

In the process of object tracking by Deep SORT, the particle movement speed of particles was relatively slow, and the motion uncertainty was low. Therefore, the association of motion information was regarded as the main matching index. λ and $A_{\max}$ took the values 0.8 and 30, respectively.

### 4.2. Dataset

A binary mixture of coarse and small particles often assumes multiple spatial distribution states. The degree of mixing is an index used to evaluate the distribution uniformity of the number of binary particles in the mixing process. Zero indicates that the coarse and small particles are completely separated, and 100 indicates that the coarse and small particles are evenly distributed. The collected images, with mixing degrees of 0, 20, 40, 60, 80, and 100, were divided into three categories according to the proportion of particles (i.e., coarse particles account for 25%, 50%, and 75%). There were 18 categories in total, each of which contained 10 images in different compaction states. We then obtained a total of 180 images of particles, and each image of particles was cropped to make the effective data in the image more obvious. Because of insufficient data, we used image enhancement technology (e.g., translation, rotation, and affine transformation) so that each image could increase the other four additional images. Finally, 900 images were utilized for subsequent training and analysis. Specifically, we used the LabelMe image annotation tool to generate a file in .json format and then divided the dataset into a training set, validation set, and test set at a ratio of 8:1:1.

### 4.3. Algorithm Evaluation Index

To evaluate the effects of Mask R-CNN comprehensively and objectively, we utilized the accuracy, precision, recall, and F1 score as algorithm evaluation indicators.

Accuracy is the percentage of correct prediction results in the total samples, and it is calculated using Equation (13):

(13) $$Accuracy=\frac{T_{P}+T_{N}}{T_{P}+F_{P}+T_{N}+F_{N}}$$

where $T_{P}$ is the number of pixels that actually belong to particles and are predicted to be particles by the model, $F_{P}$ is the number of pixels that actually belong to the background but are predicted to be particles by the model, $T_{N}$ is the number of pixels that actually belong to the background and are predicted to be background by the model, and $F_{N}$ is the number of pixels that actually belong to particles but are predicted to be background by the model. Therefore, $T_{P}+T_{N}$ is the number of pixels correctly predicted by the model, and $F_{P}+F_{N}$ is the number of pixels incorrectly predicted by the model, where $T_{P}+F_{P}+T_{N}+F_{N}$ is the total number of pixels.

Precision is the ratio of the number of samples that are correctly identified as a certain class to the actual number of this class. Recall is the ratio of the number of samples that are correctly identified as a certain class to the predicted total number of this class. These are shown in the following equations:

(14) $$Precision=\frac{TP}{TP+FP}$$

(15) $$Recall=\frac{TP}{TP+FN}$$

where *TP* is the number of samples that are actually positive and divided into positive classes by the classifier, *FP* is the number of samples that are actually negative and divided into positive classes by the classifier, and *FN* is the number of samples that are actually positive and divided into negative classes by the classifier.

The F1 score is the harmonic mean of accuracy and recall, and the calculation formula is as follows:

(16) $$F1=2\times \frac{Precision\times Recall}{Precision+Recall}.$$

If the objects switched frequently in the process of tracking the movement of rock particles using Deep SORT, the tracking effect would be worse. Therefore, we operated the ID switching rate ($ID_{switch}$) of the objects as the evaluation index of the tracking effect of the rock particles. $ID_{switch}$ can be expressed as follows:

(17) $$ID_{switch}=\frac{I_{switch}}{I_{all}}.$$

where $I_{switch}$ is the number of IDs switching during the tracking, and $I_{all}$ is the total number of detected objects in the video stream.

We utilized the detection errors $\delta_{T}$ and $\delta_{R}$ to indicate the accuracy of translation and rotation in a video. These calculation methods are shown in Equations (18) and (19):

(18) $$\delta_{T}=\frac{1}{PF}\sum_{p=1}^{P}\sum_{f=1}^{F}\frac{\left|T_{p}^{f}-t_{p}^{f}\right|}{t_{p}^{f}},$$

(19) $$\delta_{R}=\frac{1}{PF}\sum_{p=1}^{P}\sum_{f=1}^{F}\frac{\left|R_{p}^{f}-r_{p}^{f}\right|}{r_{p}^{f}},$$

where *P* is the number of particles selected to calculate the detection errors, which is set to 10 in this experiment. F is the number of images selected to calculate the detection errors in the video. Moreover, we selected an image every 5 s (150 frames) in this experiment. $T_{p}^{f}$ and $R_{p}^{f}$ respectively represent the translation and rotation detected by the visual method of the *p-th* particle in the *f-th* image, with $t_{p}^{f}$ and $r_{p}^{f}$ respectively being the true translation and rotation of the *p-th* particle in the *f-th* image.

### 4.4. Instance Segmentation Experiment

To verify the effectiveness of the improved Mask R-CNN for the detection and segmentation of rock particles, we compared it with the standard Mask R-CNN and other state-of-the-art methods. Firstly, we calculated detection and segmentation performance evaluation indicators on the self-created dataset and recorded them in Table 2. Secondly, we reported instance segmentation results on COCO using the standard metrics in Table 3. All models were trained on the *train2017* and tested on the *val2017*. Final results were on *test-dev*. Finally, we also compared the improved results of the classification loss with some enhancement results of Mask R-CNN on COCO *minival* (Table 4).

**Figure 7:**
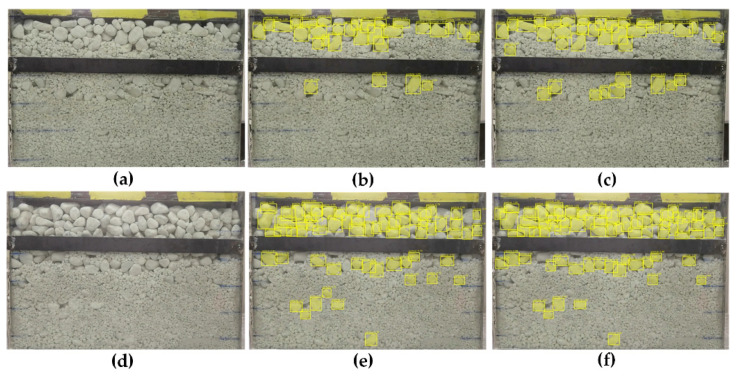
Experiment effect images of instance segmentation: (**a**) shows an image with a mixing degree of 40, and the proportion of coarse particles is 25; (**b**) shows the segmentation effect of (**a**) with the standard Mask R-CNN; (**c**) shows the segmentation effect of (**a**) with the improved Mask R-CNN; (**d**) shows an image with a mixing degree of 60, and the proportion of coarse particles is 50; (**e**) shows the segmentation effect of (**d**) with the standard Mask R-CNN; (**f**) shows the segmentation effect of (**d**) with the improved Mask R-CNN.

The original images of particles are shown in Figure 7a,d. Figure 7b,e show the detection and segmentation results of the rock particles by the Mask R-CNN. Some rock particles were not detected. For particles largely obscured and surrounded by small particles, the missing detection is more serious. This means that it is difficult to classify coarse particles with a high degree of similarity in the backgrounds, which results in a poor detection effect. The segmented mask can cover the object, and the edge contour of the coarse particles does not have obvious undersegmentation or oversegmentation. Figure 7c,f show the results of the improved Mask R-CNN. After adding the classification loss function based on Arcface loss, the Mask R-CNN has a certain improvement in the detection effect of rock particles, which means that it can detect and segment some rock particles that are partially covered or surrounded by small particles. The segmentation effect is similar to the previous standard Mask R-CNN algorithm. More particles cannot be detected as the degree of mixing and the proportion of coarse particles increase.

Table 2 shows that all indicators have been increased through the improved Mask R-CNN to detect and segment the rock particles. Recall has the largest increase of 5.99%, which indicates that the algorithm has improved in distinguishing the particles and backgrounds in pictures, and more objects can be detected. The comprehensive evaluation indexes accuracy and F1 score increased by 4.20% and 4.28%, respectively. This shows that the overall detection and segmentation effect of the improved algorithm is better than that of the standard algorithm. In addition, the improved Mask R-CNN also shows better results than other state-of-the-art methods. The improved Mask R-CNN is more suitable for subsequent VIS, ellipse characterization, and detection of motion information of particles.

As shown in Table 3, the improved Mask R-CNN offers competitive instance segmentation performance. The instance segmentation results of the improved Mask R-CNN on COCO are slightly better than the standard Mask R-CNN. Table 3 also reports a certain mask AP improvement compared to FCIS, RetinaMask, and YOLACT. Our improvement to Mask R-CNN also has some advantages on public datasets.

Table 4 shows that all improvements increase mask AP by 2.9 points (from 36.7 to 39.6) and box AP by 4.4 points (from 39.6 to 44.0). The addition of the classification loss function increases both mask AP and box AP. This shows that the new loss function can be used as a component to improve the detection and segmentation effect of Mask R-CNN.

### 4.5. VIS Experiment

**Figure 8:**
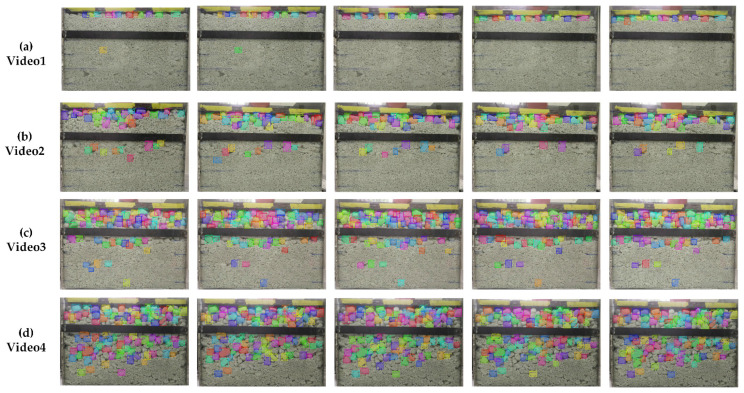
Experiment effect of VIS: (**a**) shows the tracking effect of video1 with a mixing degree of 80, and the proportion of coarse particles is 25; (**b**) shows the tracking effect of video2 with a mixing degree of 40, and the proportion of coarse particles is 25; (**c**) shows the tracking effect of video3 with a mixing degree of 60, and the proportion of coarse particles is 50; (**d**) shows the tracking effect of video4 with a mixing degree of 60, and the proportion of coarse particles is 75.

We also processed four videos to verify the efficacy of the improved Mask R-CNN and Deep SORT. The fps of the videos was 30, and each video contained a complete compaction process. As shown in Figure 8, we selected five frames with obvious changes from each video as qualitative experimental results to reflect the VIS effect during vibrational compaction. The tracking evaluation indicators of the particles were measured and are listed in Table 5. 

Figure 8 shows that the combination of the improved Mask R-CNN and Deep SORT can realize the VIS of rock particles. The improved Mask R-CNN can detect and segment objects frame by frame, and then we input the bounding box into Deep SORT to achieve object tracking. This experiment could process the video stream and integrate the detection, segmentation, and tracking of rock particles to achieve VIS, which provided the basis of machine vision for motion information detection of particles.

Table 5 lists the information statistics of the video stream. *ID_switch_* is low, which shows that the switching probability of the particle ID is low, and its tracking effect is significant. The *ID_switch_* of the improved Mask R-CNN and Deep SORT are lower than that of standard Mask R-CNN, which indicates that better detection and segmentation benefit the tracking quality. We also found the *ID_switch_* was improved even more for the videos with higher degree of mixing and proportion of coarse particles. Videos with more coarse particles are easier to switch IDs. This means that complex and crowded scenes may cause difficulty in information correlation in tracking, which negatively influences the tracking effect of the Deep SORT.

### 4.6. Particle Motion Information Detection Experiment

**Figure 9:**
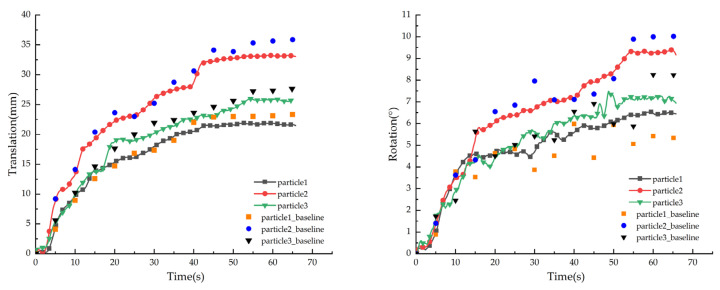
Motion information of some rock particles.

Based on the improved Mask R-CNN and Deep SORT, we measured the translation and rotation of coarse particles. The data collection frequency was high (30 times per second) because the videos were analyzed frame by frame. We used motor vibrations to produce translation and rotation so that translation and rotation curves had several jitters with small amplitudes and high frequencies. Therefore, the Savitzky–Golay filter, which can filter high-frequency jitter without changing the shape of the curve, was used to process the curve. The filtered translation and rotation curves of some particles are shown in Figure 9.

We developed a method to extract the translation and rotation of particles by manually marking the long axis to compare the effects of the machine vision method. We selected a frame every 5 s from the videos and used LabelMe to manually label the long axes of selected particles. Then, the long axes coordinates were extracted from the .json file generated by the annotation. The translation and rotation of particles were calculated using the long axes coordinates. As shown in Figure 9, the translation and rotation extracted by this method were taken as real values and were used to construct a scatter diagram. To further verify the accuracy of the vision method proposed in this study, we used Equations (18) and (19) to calculated the detection errors *δ_T_* and *δ_R_* (Table 6).

As shown in Figure 9, the translation and rotation of rock particles increase with time under the vibration load. The long axes of the equivalent ellipses are difficult to determine for particles with shapes close to the standard circle. Therefore, the detection errors for rotation are greater than those for translation (Table 6). We found that the improvement of the Mask R-CNN algorithm does not significantly reduce the detection error (only 0.12% and 0.69%). However, the improvement of Mask R-CNN reduced the ID switching rate, which is important for the tracking effects of particles. The analysis is presented below.

**Figure 10:**
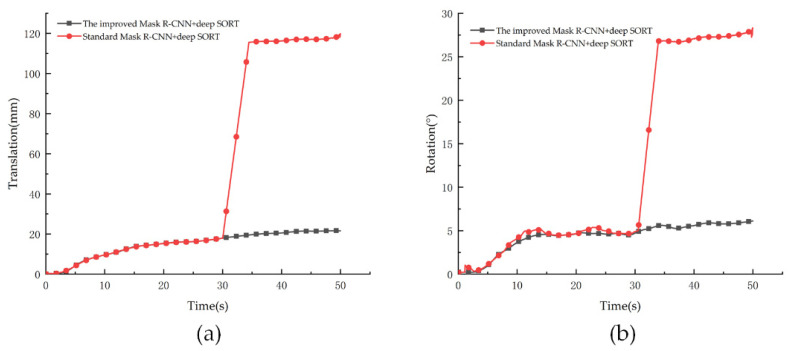
The example of the failure of standard Mask R-CNN and Deep SORT. (**a**) is the translation comparison chart. (**b**) is the rotation comparison chart.

Figure 10 shows examples of the standard Mask R-CNN and Deep SORT failures. Figure 10a shows the translation curve of the standard and improved algorithms and Figure 10b shows the rotation curve. As the particle tracked by the standard Mask R-CNN and Deep SORT switched ID is in the range of 30–35 s, the translation and rotation of the particle changed dramatically during this time. In contrast, the tracking curves of the improved Mask R-CNN and Deep SORT remained stable. The improved Mask R-CNN could reduce the ID switching rate, although it did not significantly improve the segmentation effect. Therefore, the improvement in Mask R-CNN was beneficial to the tracking effect and accuracy of detection because any ID switch would have a significant impact on the tracking result.

### 4.7. Discussion

The motion information of rock particles under a vibration load is related to the vibration amplitude and frequency as well as the shape, position, and material characteristics of the particles themselves. This experiment can detect the translation and rotation of many particles based on VIS.

The thickness of the transparent panel was 10 mm. The particles in the middle are closer to the lens because the transparent panel near the center is under more pressure, which might cause the translation of middle particles to be greater than the actual translation. The deformation of the transparent panel was 3–6 mm by measurement, and the overall movement range of the particles in this experiment was small, so we think this error could be ignored. In addition, the deformation of the transparent panel may change the light path and lens distortion, but the errors of translation and rotation will not be particularly large because they are calculated by relative position.

The detection of rock particle motion information proposed in this study has the following three limitations. First, the machine vision method cannot detect the internal and occluded particles. Second, this method approximately converts a three-dimensional space to a two-dimensional space, which adversely affects the test results. Finally, this method causes particle ID switching during the tracking process and this can significantly affect the tracking process, even though the probability of switching is low.

## 5. Conclusions and Outlook

We proposed a method for detecting the motion information of rock particles under a vibration load based on VIS. First, we improved the Mask R-CNN and designed a classification loss function based on Arcface loss to improve feature discrimination. The method can adapt to the phenomenon of high similarity between objects and backgrounds in the experimental environment. The combination of the improved Mask R-CNN and Deep SORT can achieve multiparticle VIS. Finally, we used the equivalent ellipse characterization method for segmented particles. Our method can combine with the proportional calibration algorithm to obtain the translation and rotation of particles by calculating the change in angle of the long axis of the characterizing ellipse. Experimental results show that the improved Mask R-CNN is better than the standard algorithm. The improved Mask R-CNN can perform VIS by combining it with a Deep SORT. The ID switching rate is low, and the motion information of the rock particles can be detected successfully. This is a foundation for researching the movement law and quantitative analysis of particles under a vibration load. In the follow-up, we will continue to research the motion information detection of small particles and analyze the detection results.

## Figures and Tables

**Table 1:** Key training parameters.

Parameter Type	Parameter	Value
Global Parameter	Number of categories	2
	Initial weight	1
	Learning rate	10^−3^
	Attenuation coefficient of learning rate	10^−4^
	Attenuation step length	100
	Momentum Factor	0.9
RPN Mesh Parameter	Anchor size	16, 32, 64, 128, 256
	Anchor shape ratio	0.5, 1, 2
	Anchor step length	1
	Maximum number of Anchors per image	256
	Non-Maximum Suppression (NMS) threshold for anchor	0.7
	The number of anchors after NMS	2000
Header Network Parameter	Confidence threshold	0.7
	Maximum number of instances	100
	NMS threshold	0.3

**Table 2:** Experimental results of instance segmentation on the self-created dataset.

Method	*Accuracy*%	*Precision*%	*Recall*%	*F*1%
FCIS [42]	71.97	76.26	59.68	66.96
RetinaMask [43]	86.34	85.71	69.53	76.78
YOLACT [44]	73.28	74.45	60.11	66.52
Standard Mask R-CNN	89.16	89.72	72.17	79.99
Improved Mask R-CNN	**93.36**	**91.41**	**78.16**	**84.27**

**Table 3:** The instance segmentation results on COCO.

Method	$AP_{}^{m}$	$AP_{50}^{m}$	$AP_{75}^{m}$
FCIS [42]	29.5	51.5	30.2
RetinaMask [43]	34.7	55.4	36.9
YOLACT [44]	29.8	48.5	31.2
Standard Mask R-CNN	35.7	58.5	37.8
Improved Mask R-CNN	**36.0**	**58.9**	**38.3**

**Table 4:** Comparison of the improved results of the classification loss with some enhancement results of Mask R-CNN on COCO *minival*. All the results use ResNet-101 as the backbone. Each row adds an extra component to the above row. The Mask R-CNN results are from Table 8 in the Appendix of Mask R-CNN [26] (Copyright Year: 2017. Copyright Owner’s Name: Kaiming He).

Method	$AP_{}^{m}$	$AP_{50}^{m}$	$AP_{75}^{m}$	$AP_{}^{bb}$	$AP_{50}^{bb}$	$AP_{75}^{bb}$
Standard Mask R-CNN	36.7	59.5	38.9	39.6	61.5	43.2
+ update baseline	37.0	59.7	39.0	40.5	63.0	43.7
+ c2c training	37.6	60.4	39.9	41.7	64.1	45.2
+ ImageNet–5k	38.6	61.7	40.9	42.7	65.1	46.6
+ train-time augmentation	39.2	62.5	41.6	43.5	65.9	47.2
+ loss function (ours)	**39.6**	**63.1**	**42.3**	**44.0**	**66.5**	**47.7**

**Table 5:** The tracking evaluation indicators of the particles. “SM + D” is the experimental results of the standard Mask R-CNN and Deep SORT. “IM + D” represents the experimental results of the improved Mask R-CNN and Deep SORT.

Video	Method	*I_switch_*	*I_all_*	*ID_switch_*%
Video1	SM + D	73	25621	0.28
Video1	IM + D	45	25621	**0.18**
Video2	SM + D	1059	59549	1.78
Video2	IM + D	638	59549	**1.07**
Video3	SM + D	3554	191257	1.86
Video3	IM + D	2811	191257	**1.47**
Video4	SM + D	3977	142534	2.79
Video4	IM + D	2722	142534	**1.91**

**Table 6:** The detection errors. “SM + D” and “IM + D” are the same as in Table 2. No ID switching occurs for the 10 selected particles in each video.

Video	Method	*δ_t_*%	*δ_r_*%
Video1	SM + D	3.53	**11.84**
Video1	IM + D	**3.47**	12.31
Video2	SM + D	5.51	15.43
Video2	IM + D	**5.14**	**13.81**
Video3	SM + D	**6.27**	**15.28**
Video3	IM + D	6.41	16.06
Video4	SM + D	5.58	18.16
Video4	IM + D	**5.36**	**15.79**
Average	SM + D	5.22	15.18
Average	IM + D	**5.10**	**14.49**

## Data Availability

Data sharing not applicable.

## References

[1] Jerónimo P., Resende R., Fortunato E. (2020). An assessment of contact and laser-based scanning of rock particles for railway ballast. Transp. Geotech..

[2] Zhou Y., Shi Z., Zhang Q., Jang B., Wu C. (2019). Damming process and characteristics of landslide-debris avalanches. Soil Dyn. Earthq. Eng..

[3] Gao G., Meguid M.A., Chouinard L.E., Xu C. (2020). Insights into the Transport and Fragmentation Characteristics of Earthquake-Induced Rock Avalanche: Numerical Study. Int. J. Géoméch..

[4] Kh A.B., Mirghasemi A.A., Mohammadi S. (2011). Numerical simulation of particle breakage of angular particles using combined DEM and FEM. Powder Technol..

[5] Liu G.Y., Xu W.J., Sun Q.C., Govender N. (2020). Study on the particle breakage of ballast based on a GPU accelerated discrete element method. Geosci. Front..

[6] Bagherzadeh H., Mansourpour Z., Dabir B. (2021). Numerical analysis of asphaltene particles evolution and flocs morphology using DEM-CFD approach. J. Pet. Sci. Eng..

[7] Li B., Liang Y., Zhang L., Zou Q. (2019). Breakage law and fractal characteristics of broken coal and rock masses with different mixing ratios during compaction. Energy Sci. Eng..

[8] Yang L., Fan Y., Xu N. Video instance segmentation. Proceedings of the IEEE International Conference on Computer Vision (ICCV).

[9] Yen Y.K., Lin C.L., Miller J.D. (1998). Particle overlap and segregation problems in on-line coarse particle size measurement. Powder Technol..

[10] Zhang G.Y., Liu G.Z., Zhu H. (2011). Segmentation algorithm of complex ore images based on templates transformation and reconstruction. Int. J. Miner. Metall. Mater..

[11] Amankwah A., Aldrich C. Automatic ore image segmentation using mean shift and watershed transform. Proceedings of the 21st International Conference Radioelektronika (RADIOELEKTRONIKA).

[12] LeCun Y., Bengio Y., Hinton G. (2015). Deep learning. Nature.

[13] Yuan L., Duan Y. A Method of Ore Image Segmentation Based on Deep Learning. Proceedings of the International Conference on Intelligent Computing (ICIC).

[14] Duan J., Liu X., Wu X., Mao C. (2019). Detection and segmentation of iron ore green pellets in images using lightweight U-net deep learning network. Neural Comput. Appl..

[15] Liu X., Zhang Y., Jing H., Wang L., Zhao S. (2020). Ore image segmentation method using U-Net and Res_Unet convolutional networks. RSC Adv..

[16] Jaboyedoff M., Oppikofer T., Abellán A., Derron M.H., Loye A., Metzger R., Pedrazzini A. (2012). Use of LIDAR in landslide investigations: A review. Nat. Hazards.

[17] Scaioni M. (2015). Modern Technologies for Landslide Monitoring and Prediction.

[18] Adrian R.J. (1991). Particle-imaging techniques for experimental fluid-mechanics. Annu. Rev. Fluid Mech..

[19] Sutton M.A., Mcneill S.R., Helm J.D., Chao Y.J. (2000). Advances in two-dimensional and three-dimensional computer vision. Top. Appl. Phys..

[20] Bing P., Wu D., Xia Y. (2012). Incremental calculation for large deformation measurement using reliability-guided digital image correlation. Opt. Lasers Eng..

[21] Stanier S.A., Blaber J., Take W.A., White D. (2016). Improved image-based deformation measurement for geotechnical applications. Can. Geotech. J..

[22] Travelletti J., Malet J.P. (2012). Characterization of the 3d geometry of flow-like landslides: A methodology based on the integration of heterogeneous multi-source data. Eng. Geol..

[23] Gance J., Malet J.P., Dewez T., Travelletti J. (2014). Target detection and tracking of moving objects for characterizing landslide displacements from time-lapse terrestrial optical images. Eng. Geol..

[24] Shum H., Komura T. Tracking the translational and rotational movement of the ball using high-speed camera movies. Proceedings of the IEEE International Conference on Image Processing (ICIP).

[25] Guccione D.E., Thoeni K., Giacomini A., Buzzi O., Fityus S. (2020). Efficient Multi-View 3d Tracking of Arbitrary Rock Fragments upon Impact. Int. Arch. Photogramm. Remote Sens. Spat. Inf. Sci..

[26] He K., Gkioxari G., Dollár P., Girshick R. Mask r-cnn. Proceedings of the IEEE International Conference on Computer Vision (ICCV).

[27] Ren S., He K., Girshick R., Sun J. (2017). Faster R-CNN: Towards Real-Time Object Detection with Region Proposal Networks. IEEE Trans. Pattern Anal. Mach. Intell..

[28] Li D., Chen Y., Zhang K., Li Z. (2019). Mounting behaviour recognition for pigs based on deep learning. Sensors.

[29] Fang X., Jie W., Feng T. (2019). An industrial micro-defect diagnosis system via intelligent segmentation region. Sensors.

[30] Li X., Fan Z., Liu Y., Li Y., Dai Q. (2019). 3d pose detection of closely interactive humans using multi-view cameras. Sensors.

[31] Wang Y., Li H., Jia P., Zhang G., Wang T., Hao X. (2019). Multi-Scale DenseNets-Based Aircraft Detection from Remote Sensing Images. Sensors.

[32] Long J., Shelhamer E., Darrell T. Fully convolutional networks for semantic segmentation. Proceedings of the IEEE Conference on Computer Vision and Pattern Recognition (CVPR).

[33] Liu W., Wen Y., Yu Z., Yang M. Large-margin softmax loss for convolutional neural networks. Proceedings of the 33rd International Conference on Machine Learning (ICML).

[34] Liu W., Wen Y., Yu Z., Li M., Raj B., Song L. Sphereface: Deep hypersphere embedding for face recognition. Proceedings of the IEEE Conference on Computer Vision and Pattern Recognition (CVPR).

[35] Deng J., Guo J., Xue N., Zafeiriou S. Arcface: Additive angular margin loss for deep face recognition. Proceedings of the IEEE Conference on Computer Vision and Pattern Recognition (CVPR).

[36] Bewley A., Ge Z., Ott L., Ramos F., Upcroft B. Simple online and realtime tracking. Proceedings of the 2016 IEEE International Conference on Image Processing (ICIP).

[37] Wojke N., Bewley A., Paulus D. Simple online and realtime tracking with a deep association metric. Proceedings of the 2017 IEEE International Conference on Image Processing (ICIP).

[38] Chen Z., Han Z., Hao J., Zhu Q., Soh Y.C. (2015). Fusion of wifi, smartphone sensors and landmarks using the kalman filter for indoor localization. Sensors.

[39] Qiu Z., Zhao N., Zhou L., Wang M., Yang L., Fang H., Liu Y. (2020). Vision-based moving obstacle detection and tracking in paddy field using improved yolov3 and deep SORT. Sensors.

[40] Hamzeloo E., Massinaei M., Mehrshad N. (2014). Estimation of particle size distribution on an industrial conveyor belt using image analysis and neural networks. Powder Technol..

[41] Yang Z., Yuan Y., Zhang M., Zhao X., Tian B. (2019). Safety distance identification for crane drivers based on mask r-cnn. Sensors.

[42] Li Y., Qi H., Dai J., Ji X., Wei Y. Fully convolutional instance-aware semantic segmentation. Proceedings of the IEEE Conference on Computer Vision and Pattern Recognition (CVPR).

[43] Fu C.Y., Shvets M., Berg A.C. (2019). RetinaMask: Learning to predict masks improves state-of-the-art single-shot detection for free. arXiv.

[44] Bolya D., Zhou C., Xiao F., Lee Y.J. Yolact: Real-time instance segmentation. Proceedings of the IEEE/CVF International Conference on Computer Vision (ICCV).

